# Circulating *ESR1* mutations at the end of aromatase inhibitor adjuvant treatment and after relapse in breast cancer patients

**DOI:** 10.1186/s13058-018-0968-0

**Published:** 2018-05-16

**Authors:** Violette Allouchery, Ludivine Beaussire, Anne Perdrix, David Sefrioui, Laetitia Augusto, Cécile Guillemet, Nasrin Sarafan-Vasseur, Frédéric Di Fiore, Florian Clatot

**Affiliations:** 1 Department of Medical Oncology, Centre Henri Becquerel, 1 rue d’Amiens, 76038 Rouen cedex 1, France; 2 Normandie Univ, UNIROUEN, Inserm U1245, IRON group, Rouen University Hospital, Normandy Centre for Genomic and Personalized Medicine, Rouen, France; 3 Department of Bio-Pathology, Centre Henri Becquerel, Rouen, France; 4 Department of Gastroenterology, Rouen University Hospital, Rouen, France

**Keywords:** Early breast cancer, *ESR1* mutation, Circulating tumor DNA, Aromatase inhibitor resistance, Digital PCR

## Abstract

**Background:**

Detection of circulating *ESR1* mutations is associated with acquired resistance to aromatase inhibitor (AI) in metastatic breast cancer. Until now, the presence of circulating *ESR1* mutations at the end of adjuvant treatment by AI in early breast cancer had never been clearly established. In this context, the aim of the present study was to evaluate the circulating *ESR1* mutation frequency at the end of adjuvant treatment and after relapse.

**Methods:**

This monocentric retrospective study was based on available stored plasmas and included all early breast cancer patients who completed at least 2 years of AI adjuvant treatment and experienced a documented relapse after the end of their treatment. Circulating *ESR1* mutations (D538G, Y537S/N/C) were assessed by droplet digital PCR in plasma samples taken at the end of adjuvant treatment, at time of relapse and at time of progression under first line metastatic treatment.

**Results:**

A total of 42 patients were included, with a median adjuvant AI exposure of 60 months (range 41–85). No circulating *ESR1* mutation was detectable at the end of AI adjuvant therapy. At first relapse, 5.3% of the patients (2/38) had a detectable circulating *ESR1* mutation. At time of progression on first-line metastatic treatment, 33% of the patients (7/21) under AI had a detectable circulating *ESR1* mutation compared to none of the patients under chemotherapy (0/10). The two patients with a detectable *ESR1* mutation at relapse were treated by AI and had an increase of their variant allele fraction at time of progression on first-line metastatic treatment.

**Conclusions:**

Circulating *ESR1* mutation detection at the end of AI-based adjuvant treatment is not clinically useful. Circulating *ESR1* mutation could be assessed as soon as first relapse to guide interventional studies.

## Introduction

Aromatase inhibitors (AI) are a key treatment in post-menopausal hormone receptor positive (HR+) breast cancer (BC). Estrogen receptor (*ESR1*) mutation emergence has been recently highlighted as a frequent mechanism of acquired AI resistance in the metastatic setting, as well as a prognostic marker of poor outcome [1–3]. *ESR1* mutations are characterized by a frequency of no more than 2% in primary tumour [4] compared to 30% in metastatic tissues among AI-resistant patients [1, 3]. Furthermore, *ESR1* mutations located in four hot-spots (Y537N/S/C, D538G) count for 74% of all described mutations [5], and can be successfully detected in circulating tumour DNA (ctDNA) [6, 7].

Besides its use in the metastatic setting, AI are widely administered as adjuvant treatment of HR+ early BC (EBC) with a usual exposure of 5 years [8]. Despite this long time of exposure, data concerning circulating *ESR1* mutation emergence under adjuvant AI therapy are lacking. It has been reported that circulating *ESR1* mutations at the time of metastatic diagnosis in patients previously exposed to AI in the adjuvant setting may be detected at rates ranging from 2.6 [6] to 11.3% [1]. Until now, circulating *ESR1* mutation emergence between adjuvant treatment ending, relapse and AI re-exposure remains not clearly established.

In this context, the aim of the present study was to evaluate the *ESR1* circulating mutation frequency at the end of AI adjuvant treatment in EBC patients with a subsequent local or metastatic relapse.

## Patients and methods

### Patients

We retrospectively screened HR+ EBC patients treated from 2008 to 2014 by adjuvant AI for at least 2 years and who subsequently experienced a documented localized or metastatic relapse. Only patients with available blood sample collection during the last 6 months of AI in the adjuvant setting were included in the analysis.

### Droplet digital PCR analysis

Analyses for circulating *ESR1* mutation detection were performed blind to clinical data and using the same methods as we previously reported [2]. Complete methods are provided as supplementary data (available online). Briefly, after a first step of total plasma DNA extraction and quantification, a droplet-based ddPCR platform (Qx200® ddPCR System, Bio-rad Laboratories, Hercules, CA, USA) was used for detection of four *ESR1* mutations: Y537N, Y537S, Y537C and D538G.

### Statistical analysis

The primary objective was to determine the rate of circulating *ESR1* mutation detection at the end of AI-based adjuvant treatment. In cases with available samples, we also assessed the rate of circulating *ESR1* mutation at time of relapse and time of progression to the first-line treatment used in metastatic setting.

## Results

### Patients characteristics

**Fig. 1:**
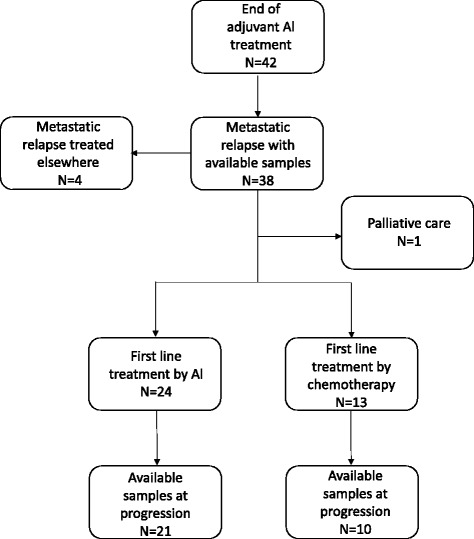
Flow chart of the study

The main characteristics of the 42 patients included are summarized in Table 1. A flow chart of the study is provided in Fig. 1. After relapse, 24 (63%) patients received AI as first-line metastatic treatment. For the remaining 14 patients, six were treated by non-AI endocrine therapy, four by chemotherapy, two by chemotherapy + anti-HER2 therapy, one by palliative care, and finally the patient with local recurrence benefited from surgery followed by adjuvant chemotherapy.

**Table 1:** Characteristics

Characteristics	N (%)	
Median age at diagnosis: years (min–max)	60.7 (47–78)	
Lymph node status	Positive	32 (76%)
	Negative	10 (24%)
Her2 status	Positive	3 (7%)
	Negative	25 (60%)
	Unknown	14 (33%)
HR status	Positive	42 (100%)
Neoadjuvant chemotherapy	Yes	4 (9.5%)
	No	38 (90.5%)
Adjuvant chemotherapy	Yes	33(78.6%)
	No	9(21.4%)
Adjuvant AI treatment	Yes	42(100%)
Tamoxifen before AI during adjuvant treatment	Yes	9 (21.4%)
	No	33 (78.6%)
Median duration of AI adjuvant treatment: months (min–max)	60.0 (41-85)	
Median delay between end of adjuvant treatment and relapse: months (min–max)	25 (2–71)	
Type of relapse	Local	1 (2.4%)
	Metastatic	41 (97.6%)
Median duration of follow-up during metastatic setting: months (min–max)	32.9 (1–75)

### Circulating *ESR1* mutational status at the end of the adjuvant phase and at progression during first-line AI in metastatic phase

**Fig. 2:**
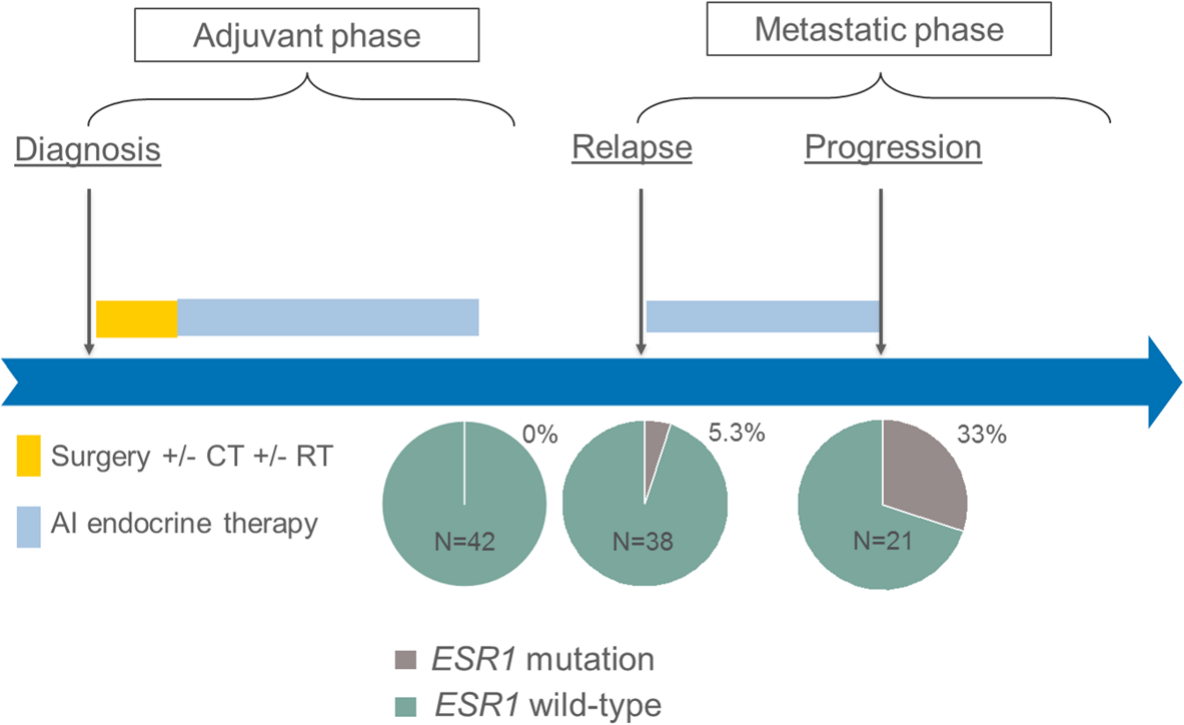
Circulating *ESR1* mutation frequency during HR+ breast cancer history. At the end of adjuvant treatment by AI, circulating *ESR1* mutations were not detected among the 42 patients of this cohort. At relapse, 2/38 patients (5.3%) had a detectable circulating *ESR1* mutation. At progression after re-exposure to AI as first-line metastatic treatment, 7/21 patients (33%) had a detectable circulating *ESR1* mutation. *AI* aromatase inhibitor, *CT* chemotherapy, *RT* radiation therapy

None of the 42 patients included had a circulating *ESR1* mutation detectable at the end of AI adjuvant therapy. Plasma samples at relapse were available for 38 patients (90%) of the full cohort. Among them, 2 (5.3%) had a detectable *ESR1* circulating mutation (Y537C and D538G) at time of relapse (Fig. 2). Plasma samples at time of progression on first-line metastatic treatment were available for 31 (74%) patients, including 21 treated with AI (Fig. 1). A circulating *ESR1* mutation was detected in 7/21 (33%) patients progressing on AI while none of the ten patients progressing on chemotherapy or non-AI endocrine treatment had a detectable circulating *ESR1* mutation. Of note, 3/42 patients had an early relapse (< 6 months) after the end of adjuvant AI. In none of these three patients was an *ESR1* mutation detected at the end of adjuvant AI or at relapse.

Concerning the two patients with a detectable circulating *ESR1* mutation at relapse after adjuvant treatment, the first had adjuvant treatment consisting of 3 years of tamoxifen followed by 4 years of AI. Metastatic relapse occurred 4 years after adjuvant treatment completion, with a Y537C mutation detected at 0.7% variant allele fraction (VAF) in circulating plasma at time of relapse. After 11 months of first-line AI, a documented clinical progression was observed with the same detectable circulating Y537C mutation, which increased significantly to 15.2% VAF.

The second patient with a detectable circulating *ESR1* mutation at first relapse received 5 years of AI adjuvant treatment and experienced a metastatic relapse at 20 months of follow-up. The circulating *ESR1* D538G mutation was detected at 6.7% VAF rate at time of relapse and increased to a 14% VAF at time to progression after 9 months under AI in the metastatic setting.

## Discussion

To our knowledge, this study is the first addressing the frequency of circulating *ESR1* mutation at the end of AI adjuvant treatment in patients treated for an EBC. Our results highlighted that no circulating *ESR1* mutation was detectable at the end of AI-based adjuvant treatment in HR+ EBC patients who also subsequently experienced a relapse during follow-up. In contrast, re-exposure to AI in the metastatic setting induced circulating *ESR1* mutation emergence among 33% of patients, which was close to the 30–50% circulating *ESR1* mutation frequency usually observed for metastatic patients progressing on AI [1–3, 9]. Interestingly, we observed that circulating *ESR1* mutation was detectable in a small proportion of patients (5.3%) at metastatic diagnosis, which is also a finding in line with previous data reporting a frequency ranging from 2.6 to 11.3% in that setting [1, 8].

Our study has some inherent limitations due to its retrospective design. First, considering the limited number of patients, the emergence of a circulating *ESR1* mutation at the end of the adjuvant phase by AI cannot be formally excluded. Thus, larger prospective studies with pre-defined sample processing are needed to confirm our results. Second, since we focused the analysis on the four main *ESR1* mutations, we cannot exclude the presence of rare circulating mutations [10]. In terms of daily practice, our results suggest that screening for a circulating *ESR1* mutation detection at the end of adjuvant AI treatment would not be of interest. In contrast, we found that *ESR1* mutations at relapse were present in 5.3% of patients and that their levels increased under AI exposure. Even rare, circulating *ESR1* mutation at relapse after an AI exposure limited to the adjuvant setting has already been reported by others [8]. Thus, early treatment change in cases of detected circulating *ESR1* mutations should be assessed by clinical trials as soon as metastatic relapse in case of previous AI exposure, and not only after AI treatment in the metastatic setting.

Concerning *ESR1* mutation emergence during the metastatic phase, it has to be noted that despite long-term exposure to adjuvant AI (median of 5 years), most of the patients with a detectable *ESR1* mutation in the metastatic setting had a detected circulating mutation only after 20 months of AI re-exposure. On the other hand, the observation that two patients harboured *ESR1* mutations at relapse confirms the hypothesis that mutational clone emergence was selected by AI therapy during the adjuvant setting, but not detectable at that time given a low tumour burden [8]. Interestingly, these two patients had a comparable delay (20 and 48 months) between end of AI adjuvant treatment and metastatic relapse compared to the other patients of the cohort (median of 25 months).

To conclude, ours results show that detection of circulating *ESR1* mutation at the end of AI-based adjuvant treatment in HR+ EBC patients is not clinically useful. Even if technical advances—such as plasmapheresis or use of implanted ultrasensitive devices [11]—may increase the amount of evaluable ctDNA and thus improve sensitivity of mutation detection in the future, ongoing efforts to detect circulating *ESR1* mutations should be focused on the metastatic setting.
